# Large and bleeding gastroduodenal artery aneurysm: Challenging diagnosis and treatment. A case report

**DOI:** 10.1016/j.ijscr.2023.109105

**Published:** 2023-12-08

**Authors:** Marco Yusef, Sara Claudia Barone, Francesco D'Angelo, Paolo Aurello, Gianfranco Silecchia, Niccolò Petrucciani

**Affiliations:** Department of Medical and Surgical Sciences and Translational Medicine, Faculty of Medicine and Psychology, St Andrea Hospital, Sapienza University, Rome, Italy

**Keywords:** Gastroduodenal artery, Emergency, Gastrointestinal endoscopy, Angiography, Endovascular treatment

## Abstract

**Introduction:**

Visceral artery aneurysms (VAA), including gastroduodenal artery aneurysms (GAA), are rare pathologies that can be challenging to diagnose due to their often-asymptomatic nature. VAA are usually correlated to atherosclerosis, fibro dysplasia, or hemodynamics changes, while pseudo aneurysms are mostly correlated to infection, inflammation, traumas, or iatrogenic lesions.

**Presentation of case:**

We report the case of an 82-years-old female presenting with abdominal pain and hematemesis. Upper gastrointestinal endoscopy retrieved a large duodenal mass and subsequent CT scans identified a large GAA with contrast extravasation. Endovascular procedure included selective arteriography, microcatheterization, and embolization.

**Discussion:**

VAA are mostly located in the splenic and hepatic artery. Symptoms of VAA are related to pressure on neighboring organs. VAA rupture is associated with a high mortality risk (over 76 %) and presents with symptoms like acute abdominal pain, hematemesis, and hemodynamic shock. Diagnosis is often made through CT scans and angiography. Treatment options for VAAs and GAAs include both surgical and endovascular methods. Endovascular treatment is preferred, with a success rate of 89 %–98 %.

**Conclusion:**

This case provides an example of challenging diagnosis and treatment of a large and bleeding GAA.

## Highlights

• Visceral artery aneurisms VAA are challenging to diagnose.
• Gastroduodenal artery aneurisms GAA are mostly symptomatic.
• VAA's treatment can be endovascular repair ER or open surgery.
• ER is less invasive and can be used in patients with higher surgical risk.

## 1 Introduction

Visceral artery aneurysms (VAA) and pseudo aneurysms are rare pathologies affecting the celiac trunk, the superior and inferior mesenteric arteries, and their branches, excluding the aortoiliac axis. [1] VAA can be found in 0.01 %–0.2 % of patients.

GAA represent 1.5 % of all VAA. GAA are located in the gastroduodenal artery (GDA), between the common hepatic artery and the right gastroepiploic artery [2], and have a rupture's risk of 25 %, occurring with gastrointestinal bleeding, leading to a 70 % risk of death [3].

VAA are usually correlated to atherosclerosis, fibro dysplasia, pancreatitis, or hemodynamics changes like portal hypertension, while pseudo aneurysms are mostly correlated to infection, inflammation, traumas, or iatrogenic lesions [4].

Normally, VAA are asymptomatic or have a non-specific clinical presentation. In contrast to the majority of VAA, GAAs tend to be symptomatic [5].

Symptoms are correlated to acute or chronic disease: acute cases may present with abdominal pain, hematemesis, hemorrhage with eventually hemorrhagic shock secondary to rupture and bleeding in the gastrointestinal tract. Chronic cases are mostly linked with anemia due the chronic blood loss [1].

This case was reported in accordance with the SCARE criteria [6].

## 2 Presentation of case

An 82-year-old female patient came to the Emergency Department with abdominal pain and hematemesis. Her vital signs were normal, and the physical examination showed abdominal pain extending to the upper quadrants with absence of abdominal distension, no objective alterations were shown performing digital rectal exam. She reported hematemesis that started 8 h before. Her body mass index (BMI) was 28 and her medical history included arterial hypertension and right breast quadrantectomy for “in situ” breast cancer. Laboratory exams showed hemoglobin 8.50 g/dl (normal value in hour hospital: 12.00–16.00 g/dl); white blood cells 15.30 ∗ 10^3/μL (normal value in hour hospital: 4.30–10.80 ∗ 10^3/μL); neutrophils percentage of 88.0 % (normal value: 45.0–74.0 %); platelets 183 ∗ 10^3/μL (normal value:140–400 ∗ 10^3/μL).

**Fig. 1:**
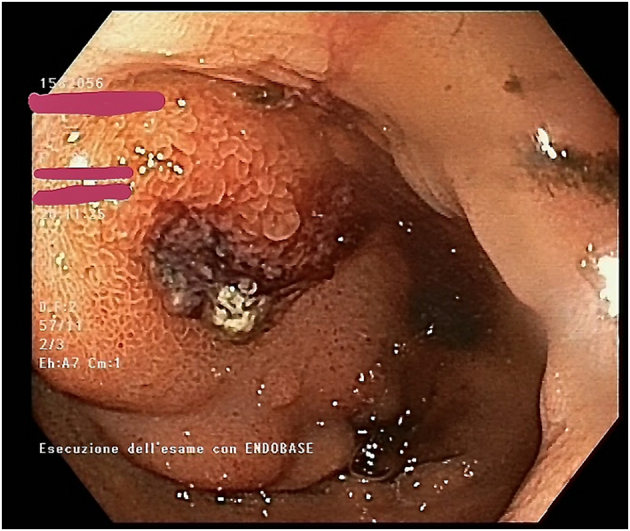
GE showing a protruding submucosal lesion in the lumen of the duodenum presenting central erosions and a visible vessel, suspect for gastrointestinal stromal tumor (GIST).

An emergency upper gastrointestinal endoscopy (GE) was performed. GE showed blood clots in the stomach and a protruding submucosal lesion in the lumen of the duodenum presenting central erosions and a visible vessel, diagnosed as a suspect gastrointestinal stromal tumor (GIST) (Fig. 1).

Metal clips were applied on the bleeding vessel and the lesion was treated with an adrenaline submucosal injection. Biopsies were not performed to avoid potential further sources of bleeding.

**Fig. 2:**
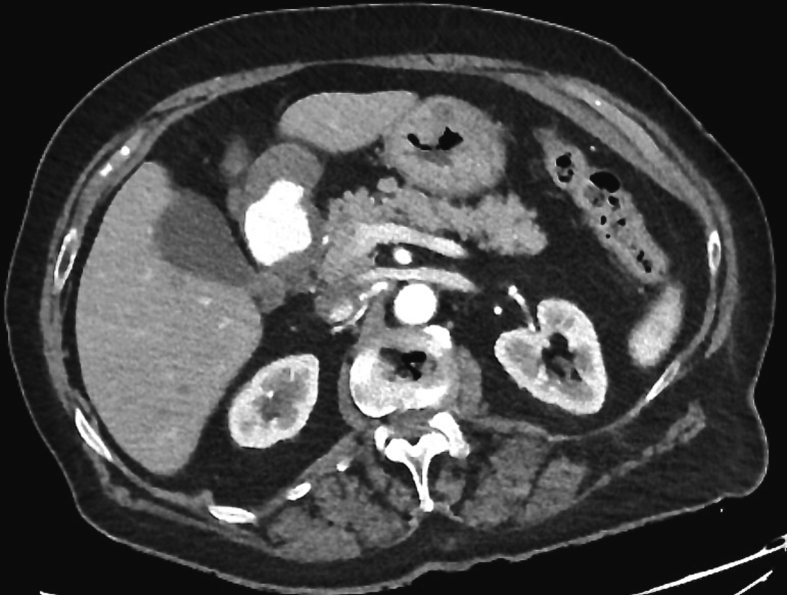
CT scan of the patient's abdomen demonstrating evident contrasts extravasation.

After GE, a computed tomography scan (CT) with intravenous contrast was performed (Fig. 2). The CT scan showed a mass both exophytic and intraparietal located between the antrum and pylorus, with evident contrasts extravasation during the arterial phase. No evidence of free air or free abdominal effusion was found in the abdominal cavity.

A diagnosis of large GAA was made.

Considering the hemodynamic stability of the patient, endovascular therapy was attempted.

Emergency selective arteriography of the celiac tripod and superselective arteriography of the hepatic artery and gastroduodenal artery were performed. Superselective catheterization of the afferent and efferent vessels was performed with a 2.9 Fr microcatheter, and embolization of the efferent vessels was performed with two 5 mm × 15 mm, controlled release coils.

The afferent vessel was embolized with 7 × 29 mm, 5 × 24 mm and 4 × 29 mm coils.

After embolization, the absence of supply of the pseudoaneurysm and closure of the vessel in the embolized tract were demonstrated.

**Fig. 3:**
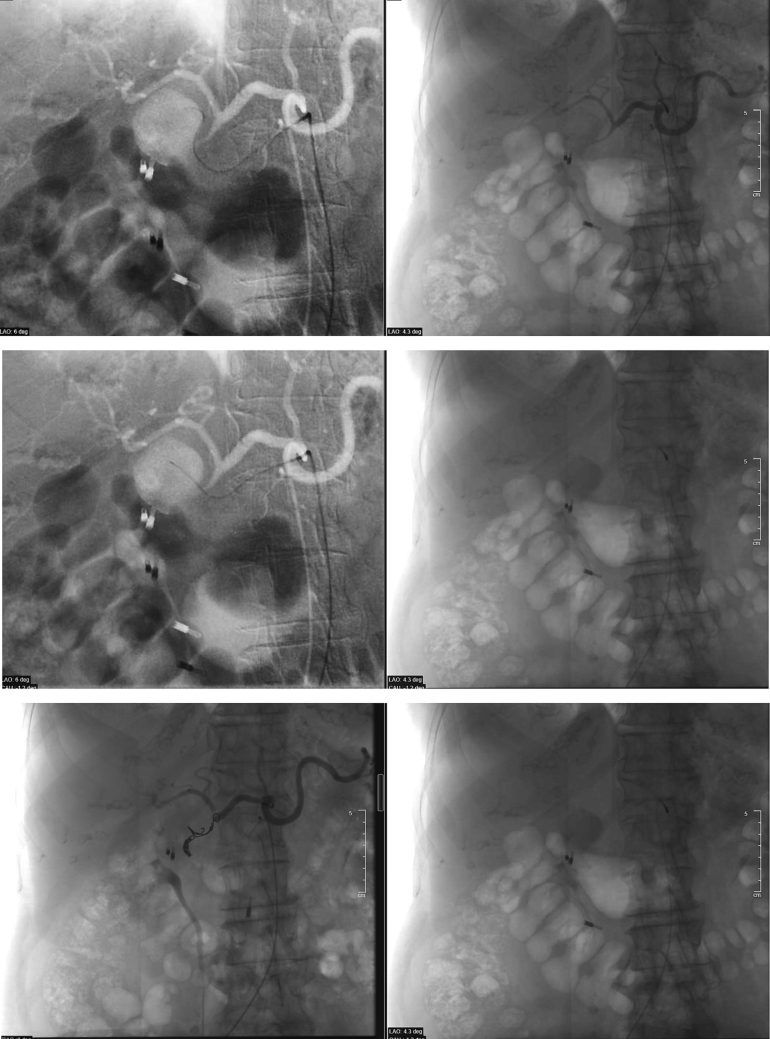
Emergency selective arteriography.

Partial migration of a coil in the common hepatic artery was detected. The common hepatic artery, however, was patent at angiography, with evidence of a small opacification defect (Fig. 3).

After the procedure the patient had two more episodes of hematemesis with preserved vital signs and hemoglobin above 10 g/dl.

**Fig. 4:**
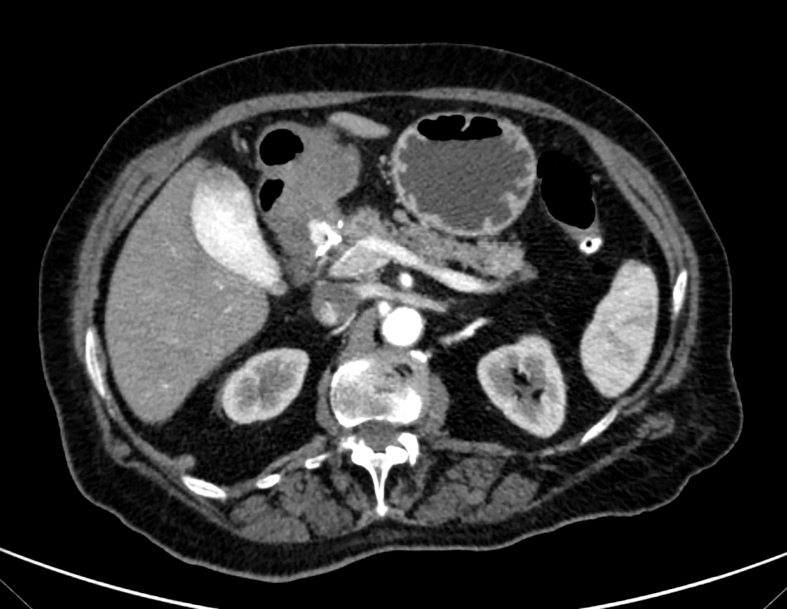
CT scan showing that the pseudoaneurysm was almost completely obliterated, with a residual small, vascularized spot of about 1 cm.

At first day after the procedure, CT scan with contrast injection showed an almost completely obliterated pseudoaneurysm. A small, vascularized spot of about 1 cm remained at the collar level (Fig. 4). The patient remained stable without new episodes of vomiting and was monitored with CT scan.

The patient was discharged at postoperative day 5 with CT scan scheduled 21 days after dismission and enoxaparin 4000 IU/day treatment for 10 days.

**Fig. 5:**
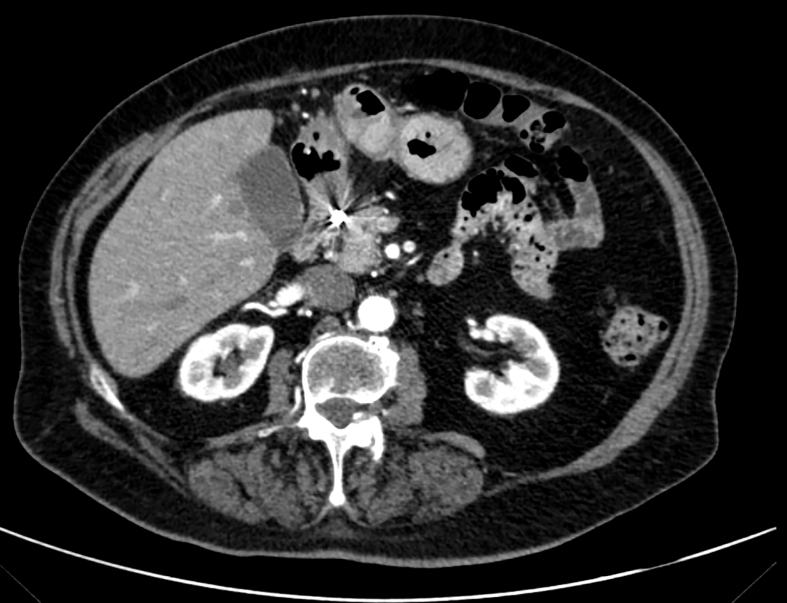
CT scan showing complete obliteration of the pseudoaneurysm of the gastroduodenal artery, with no residual sign of replenishment.

At follow-up the patient reported no symptoms, blood tests were normal, hemoglobin was stable. The CT scan showed complete thrombosis of the pseudoaneurysm of the gastroduodenal artery, with no residual sign of replenishment at the level of the collar (Fig. 5).

The aneurysm sac size was stable. No liver or other organ lesions were detected. No further follow-up was scheduled.

## 3 Discussion

VAA are mostly located in the splenic artery (40–60 % of cases) and in the hepatic artery (10–30 % of cases) while the incidence of gastroduodenal artery aneurisms is 0,01–1 % of cases.

Mostly of VAA are incidentally detected with radiological exams like CT scan, as a result, the true incidence of VAAs may be higher than what is reported in the literature.

VAA symptoms are mostly correlated to the increasing amount of pressure in the neighboring organs, that can create abdominal pain, vomiting or cholestasis. GAA tends to be symptomatic [5].

VAA rupture has a mortality risk higher than 76 % and the symptoms include acute abdominal pain, hematemesis, immediate reduction of hemoglobin and hemodynamic shock [4,10].

It is also documented that true aneurysms are associated to less symptoms and less rupture risks than false aneurysms or pseudoaneurysms.

True aneurisms pathogenesis is linked to atherosclerosis, fibrodysplasia, changes in the hemodynamics patterns like portal hypertension or celiac trunk stenosis. False aneurysms are associated to infections, inflammation, traumas or iatrogenic lesions. Alcohol abuse, cholangitis or pancreatitis can also be associated to false aneurysms.

Diagnosis of VAA is normally made by CT scan and angiography, which is used in every VAA because it can correctly establish the lesion's anatomy and it is performed in all the cases. Percutaneous angiography is also characterized by high sensitivity and the possibility of treating the patient during the same intervention. MRI and pulsed Doppler or color Doppler and three-dimensional CT can also be performed. For gastrointestinal bleeding, also endoscopy can be performed. In the present case, endoscopy was misleading as it retrieved a duodenal mass suspect as a tumoral lesion (GIST).

In emergency cases, it is advisable to stabilize the patient's vital signs and to make a correct diagnosis by CT angiography.

VAA and GAA treatment options are both endovascular treatment and open surgical repair. Endovascular treatment presents a success rate of 89 %–98 % [7], is a less invasive procedure that can be used in patients who are at higher surgical risk or when the aneurysm is in a location that is difficult to access surgically and in all the patients has a lower peri- and post-operative risk [4].

The choice of one type of embolic agent or specific techniques may vary based on the patient's condition and the location and characteristics of the aneurysm. The goal is to prevent further growth and potential rupture of the aneurysm, which can be a life-threatening event.

The first step of endovascular treatment is to isolate the aneurysm from the main artery by advancing a catheter through the arterial system and deploying detachable microcoils or other embolic agents into the vessels creating a block of the inflow. Then, proximal and distal arteries are embolized. Throughout all the procedure, imaging techniques like angiography are used to monitor the placement of coils or embolic agents and to confirm that blood flow to the aneurysm has been effectively blocked [7].

Endovascular treatment may present some risks. In fact, bleeding or infection at the catheter's access site may occur, such as blood clots, endoleaks, allergic reactions or renal damage, linked both to the contrast's dye or tissue damages.

The VAA's open surgical treatment can be done by aneurysmorrhaphy or aneurysm removal. Open treatment should be normally used if endovascular treatment can't be performed, like in emergency settings in hospitals without availability of interventional radiologists, or in cases of difficulty of catheterization, high risk of ischemia and distal embolization [8].

Endovascular treatment is usually considered the preferred treatment for VAA, although there are no randomized studies in the literature that demonstrate that this treatment is effectively superior. Rebelo et al. [9], in a retrospective single-center analysis suggests that patients who underwent endovascular treatment of VAA experienced no mortality and a shorter hospital stay, confronted to patient who underwent open surgical treatment both in elective and emergency surgery [9,10].

## 4 Conclusion

In conclusion, this case provides an excellent example of emergency diagnosis and treatment for a large and bleeding GAA. We approached this case successfully with endovascular embolization.

## Consent

Written informed consent was obtained from the patient for publication on this case report and accompanying images. A copy of the written consent is available for review by the Editor-in-Chief of this journal of request.

## Ethical approval

As no experimental treatment was performed, this study is exempt from ethical approval in our institution (La Sapienza University of Rome). The patient was treated according to the standard of care of our Hospital. Patient approval has been given.

## Funding

No sources of funding.

## Author contribution

Marco Yusef; Sara Claudia Barone; Niccolò Petrucciani; Francesco D'Angelo; Paolo Aurello;– Supervision, Data curation, Writing, Review and Editing

Sara Claudia Barone; Marco Yusef; Niccolò Petrucciani– Conceptualization, Data curation, Writing - Original draft

Gianfranco Silecchia; Niccolò Petrucciani – Supervision, Data curation, Writing, Review and Editing

All – approval of final manuscript

## Guarantor

Niccolò Petrucciani.

## Declaration of competing interest

This research did not receive any specific grant from funding agencies in the public, commercial, or not-for-profit sectors. The authors have no competing interests to declare.
